# Antioxidant intake among Brazilian adults - The Brazilian Osteoporosis Study (BRAZOS): a cross-sectional study

**DOI:** 10.1186/1475-2891-10-39

**Published:** 2011-04-25

**Authors:** Marcelo Medeiros Pinheiro, Rozana Mesquita Ciconelli, Gabriela Villaça Chaves, Luana Aquino, Claudia Ridel Juzwiak, Patrícia de Souza Genaro, Marcos Bosi Ferraz

**Affiliations:** 1 Rheumatology Division, Federal University of Sao Paulo/ Paulista School of Medicine (Unifesp/ EPM), Brazil; 2 Paulista Center for Health Economics, Unifesp/ EPM, Brazil; 3 Micronutrient Research Center, Josué de Castro Nutrition Institute, Universidade Federal do Rio de Janeiro, Brazil; 4 Department of Health, Education and Society, Nutrition Course, Unifesp/Baixada Santista Campus, Brazil; 5 Rheumatology Division, Federal University of Sao Paulo, Unifesp/ EPM, Brazil

**Keywords:** nutritional intake, dietary recall, micronutrients, antioxidant, Brazilian population, Public Health

## Abstract

**Background:**

Antioxidant nutrient intake and the lesser formation of free radicals seem to contribute to chronic diseases. The aim of the present study was to evaluate the intake profile of the main dietary antioxidants in a representative sample of the adult Brazilian population and discuss the main consequences of a low intake of these micronutrients on overall health.

**Methods:**

The sample comprised 2344 individuals aged 40 years or older from 150 cities and was based on a probabilistic sample from official data. The research was conducted through in-home interviews administered by a team trained for this purpose. Dietary intake information was obtained through 24-h recall. The Nutrition Data System for Research software program was used to analyze data on the intake of vitamins A, C and E, selenium and zinc, which was compared to Dietary Reference Intakes (DRIs). Differences in intake according to sex, anthropometrics, socioeconomic status and region were also evaluated. The SPSS statistical package (version 13) was used for the statistical analysis. P-values < 0.05 were considered significant.

**Results:**

Higher proportions of low intake in relation to recommended values were found for vitamin E (99.7%), vitamin A (92.4%) and vitamin C (85.1%) in both genders. Intake variations were found between different regions, which may reflect cultural habits.

**Conclusion:**

These results should lead to the development of public health policies that encourage educational strategies for improving the intake of micronutrients, which are essential to overall health and prevention of non-communicable diseases.

## Introduction

In the last 50 years, Brazil has gone through changes in eating habits, with a greater intake of processed foods, meat, oil and vegetable fat as well as a low consumption of fruit, vegetables and cereals [1-3]. These aspects, together with a sedentary lifestyle, have contributed toward a greater prevalence of obesity and other non-communicable chronic diseases [4] as well as higher disability and mortality rates [5], especially those related to cardiovascular disease [4-6].

Previous national data demonstrate that only 2 to 3% of total food intake corresponds to fruit, legumes and vegetables [2], which are important sources of nutrients with antioxidant functions. These percentages correspond to approximately one third of the internationally recommended intake for the prevention of non-communicable chronic diseases [7,9,11,12].

Antioxidants are substances that, at low concentrations, retard or minimize the oxidation of oxidable substrates [13]. Among the non-enzymatic antioxidant systems responsible for lesser cellular and molecular oxidative stress, vitamins A, C and E, zinc, and selenium merit particular attention [14]. Oxidative stress is a disorder of the state of equilibrium of the prooxidant/antioxidant system in intact cells, with consequent harm to lipids, proteins, carbohydrates and nucleic acid, leading to cell death [15]. More recently, some authors believe that it is not only an imbalance or a single entity, since redox of plasma GSH/GSSG is not equilibrated with the larger plasma cysteine/cystine (Cys/CySS) pool. Thus, from a mechanistic standpoint, oxidative stress may be better defined as a disruption of redox signaling and control [16,17].

A number of studies have demonstrated the association between antioxidant nutrient intake and the lesser formation of free radicals as well as aspects related to the pathogenesis and emergence of non-communicable chronic diseases [6,14,18-20]. On the other hand, none of these studies demonstrated that the supplementation with antioxidants has succeeded in reducing the rate of chronic diseases.

The aim of the present study was to describe the dietary intake of the main antioxidants in a representative sample of the adult Brazilian population and discuss the main consequences of a low intake of these micronutrients on overall health.

## Methods

A total of 2344 individuals over 40 years of age, residents of rural and urban areas of 150 cities in the country were evaluated in the present cross-sectional study. Individuals of all socioeconomic classes, degrees of schooling, skin colors and types of occupation were included in order to maintain the Brazilian population as representative [1,21]. The probabilistic sample was selected in three phases, controlled for gender, age and type of occupation [21,22].

The interviews were held at the residences of the interviewees and administered face to face. Interviewers carried out interviews seven days a week during the entire day and evening. The residences were selected randomly. Individuals with cognitive disabilities (neurological diseases and dementia) who could provide dubious information were excluded from the study. All of the other diseases, including hypertension and diabetes, as well as concomitant medications and lifestyle habits (smoking, physical activity and alcohol intake) were not considered as exclusion criteria. Since these conditions may modify the needs of antioxidants, several statistical adjustements were performed to correct the possible confounders. Moreover, patients taking calcium and vitamin D as well as multivitamin or other dietary supplements were excluded of this nutritional analysis.

Anthropometric evaluation involved the determination of weight (kg) and height (m) using a portable digital scale and metric tape, respectively. The body mass index (BMI) was calculated by body weight divided by height squared (kg/m^2^). The classification of nutritional state was determined based on the criteria proposed by the World Health Organization [23].

Food intake was evaluated based on 24-h recall. The interviewers were trained by a nutritionist specialized in the application of eating habit surveys. The questionnaires of individuals with a calorie intake below 400 Kcal or above 5500 Kcal were excluded. The data were analyzed with the aid of the Nutrition Data System for Research software program [24], which uses the food composition table of the US Department of Agriculture (USDA) as its database. Dietary energy and micronutrient intake was then evaluated in relation to antioxidant function, such as vitamins A, C and E, selenium, and zinc. Food supplement intake was considered and used in the final evaluation. Data on macronutrients and micronutrients related to bone health have recently been published [22,25].

For all analyses, adjustments for the energy of the diet were made based on the residual nutrient method proposed by Willett and Stampfer [26]. Dietary intake was analyzed considering the different characteristics of the population: gender, age, nutritional state, physical activity levels, region and socioeconomic status in order to correct possible confounders. Furthermore, all questionnaires were revised by an independent supervisor and underwent continuous process of criticism and consistency. Inconsistently filled questionnaires were returned for correction. About 25% of the questionnaires were verified *in loco *or *post hoc *by phone call. The Dietary Reference Intakes (DRIs) [8-10] were used as the reference values, considering age and gender.

All participants received an explanation of the goals and outcomes of this study and signed terms of informed consent. The study received approval from the Ethics Committee of the Universidade Federal de São Paulo/ Escola Paulista de Medicina, Brazil.

Statistical analyses were performed using the SPSS statistical package for Windows (version 13.0). The descriptive data were expressed as mean and standard deviation for numerical variables and percentage for qualitative variables. The Student's t-test was used for the comparison of numerical variables between groups. Analysis of variance (ANOVA) was used for multiple comparisons of numerical variables between three or more groups. When significant differences were found in the numerical variables between three or more groups, Tukey test was applied for each pair of variables separately in order to identify what levels differed between one another by calculating the adjusted *p*-value. The Kolmogorov-Smirnov test was used in all analyses to ensure the normal distribution of the nutrients. Either the chi-square test or Fisher's exact test was used to determine associations between categorical variables. For all analyses, *p*-values less than 0.05 were considered statistically significant.

## Results

The sample was made up of 2344 individuals, 693 (29.6%) of whom were men and 1651 (70.4%) of whom were women. Mean age was 58.4 ± 12.8 and 60.1 ± 13.7 years for males and females, respectively. The largest portion of the individuals was over 51 years of age and was therefore at greater risk of non-communicable chronic diseases, especially osteoporosis. Geographic and socioeconomic distributions simulated the official data of the Brazilian Institute of Geography and Statistics [1] and National Home Sampling Survey [21]. Based on nutritional state (determined by the BMI), there was a high frequency of individuals with overweight and obesity (57.5%), particularly in the male gender (Table 1). There were no significant differences in nutritional state in relation to socioeconomic class or region of the country.

**Table 1:** General characteristics of Brazilian adults

	Total (%)	Men (%)	Women (%)
**Age group**			
40-50 years	799 (34.3)	247 (35.8)	552 (33.7)
51-70 years	895 (38.4)	273 (39.4)	622 (37.7)
70 years or more	634 (27.2)	169 (24.4)	464 (28.1)
**Geographic region**			
North	307 (13.1)	94 (14.0)	210 (12.7)
Northeast	491 (20.9)	139 (20.1)	352 (21.3)
Central-West	338 (14.4)	102 (14.7)	236 (14.3)
Southeast	824 (35.1)	240 (34.6)	584 (35.4)
South	385 (16.4)	115 (16.6)	269 (16.3)
**Socioeconomic Class**			
A-B (highest)	321(13.8)	111 (16.1)	210 (12.8)
C	753 (32.4)	233 (33.9)	519 (31.4)
D-E (lowest)	1252 (49.4)	344 (49.6)	562 (34.0)
**Nutritional State**			
Malnutrition	66 (2.9)	16 (2.5)	50 (3.0)
Normality	887 (39.4)	247 (37.1)	640 (40.4)
Overweight	840 (37.3)	281 (42.3)	558 (35.2)
Obesity	459 (20.4)	121 (18.2)	338 (21.3)

Mean (Standard Deviation)

With the exception of vitamin C there were significant differences in intakes of all nutrients between genders. Men had a higher mean intake of vitamin E and zinc, while women had higher intakes of vitamin A and selenium (Table 2).

**Table 2:** Daily intake of antioxidants in Brazilian adults

Nutrients	Men	Reference value (EAR)€	Women	Reference value (EAR)€	P
Vitamin A (μg RAE)	368.7 ± 1502	625	411.3 ± 1494	500	0.02
Vitamin C (mg)	70.9 ± 536.6	75	95.4 ± 501.9	60	0.44
Vitamin E (mg)	5.1 ± 2.8	12	4.9 ± 1.9	12	0.001
Selenium (μg)	84 ± 53.4	45	84.8 ± 40.1	45	0.001
Zinc (mg)	9.6 ± 4.8	9.4	8.7 ± 3.7	6.8	0.001

Student's t-test; Mean ± Standard Deviation; RAE: Retinol Activity Equivalents; EAR^€ ^Estimate Average Requirement reference values for individuals ≥ 40 years old (8-11); adjusted for age and energy

A greater intake of selenium and vitamin A was found in the northern region. Vitamin C ingestion was significantly higher in the northeastern region. There was a greater intake of vitamin E in the central-western region, with a similar intake of these nutrients in the southeastern region. The southern and central-western regions had a greater intake of zinc (Table 3).

**Table 3:** Daily intake of antioxidants in Brazilian adults according to macro-region

Nutrients	North	Northeast	Central-West	Southeast	South	P
Vitamin A (μg RAE)	375.3 ± 2872.2*	175.6 ± 832.2	181.5 ± 898.7	258.7 ± 1156.5	256.4 ± 1288	0.036
Vitamin C (mg)	39.1 ± 195.9	162.5 ± 634.7*	95 ± 473.7	71 ± 597.7	63.6 ± 290.7	0.005
Vitamin E (mg)	4.8 ± 2.5	4.5 ± 2	5.1 ± 1.9*	5.1 ± 2.2*	5.0 ± 2.8*	0.001
Selenium (mg)	105.9 ± 79.5*	84.7 ± 40.8	76.7 ± 31.6	78.4 ± 32.2	87.8 ± 35.7	0.001
Zinc (mg)	8.6 ± 4.5	8.7 ± 4	9.4 ± 4.1*	8.8 ± 3.3	9.5 ± 5*	0.005

ANOVA; RAE: Retinol Activity Equivalents; *: higher intake values in each region and significantly different from the others; Tukey test; adjusted for age and energy

The association between antioxidant intake and nutritional state was significant only for zinc, which was higher among individuals with a normal nutritional state than those with overweight or obesity (Table 4).

**Table 4:** Daily intake of antioxidants in Brazilian adults according to nutritional state

Nutrients	Normality	Overweight	Obesity	P
Vitamin A (μg RAE)	495.1 ± 1979.6	332.9 ± 1023.6	372.2 ± 836.7	0.24
Vitamin C (mg)	83.5 ± 397.9	87.5 ± 582.7	138.5 ± 594	0.24
Vitamin E (mg)	4.8 ± 1.7	5 ± 2.1	4.9 ± 1.8	0.51
Selenium (μg)	84.9 ± 39.3	84.1 ± 38.7	85 ± 41.3	0.93
Zinc (mg)	9.1 ± 4.5	8.4 ± 2.9	8.7 ± 3.3	0.02

Mean ± Standard Deviation; ANOVA; Tukey test; adjusted for age and energy

There was a high proportion of individuals with low intake of all antioxidant nutrients, with the exception of selenium. The highest proportion of individuals with low daily intake was for vitamin E, followed by vitamin A. A greater proportion of men had a low intake of zinc and selenium. Although we found a similar mean intake of vitamin C for men and women as shown in Table 2, the proportion of men with lower intake was significantly higher (Table 5).

**Table 5:** Proportion of Brazilian adults with low antioxidant intake based on DRI recommendations for gender

Nutrients	Total (%)	Men (%)	Women (%)	P
Vitamin A	92.4	93.4	92.1	0.37
Vitamin C	85.1	88.3*	83.7	0.05
Vitamin E	99.7	99.6	99.8	0.28
Selenium	13.4	18*	11.5	0.01
Zinc	52.1	67.4*	45.6	0.03

ANOVA; Tukey test; adjusted for age and energy; *: Significantly higher proportion of low intake

There was no statistically significant difference in the proportion of individuals with low dietary intake of vitamin A, vitamin E, and zinc in relation to region of the country. Although mean intake was below that recommended by the DRIs, there was a lower proportion of individuals with low vitamin C intake in the northeastern region. There is a lower proportion of individuals with low selenium intake in the entire country, especially in the southern region (p < 0.05) (Table 6).

**Table 6:** Proportion of Brazilian adults with low antioxidant intake according to geographic region

Nutrients	North (%)	Northeast (%)	Central-West (%)	Southeast (%)	South (%)	P
Vitamin A	94.8	90.8	94.7	92.1	91.4	0.12
Vitamin C	90.6	79.8*	85.8	85.6	85.7	0.04
Vitamin E	99	100	100	99.6	99.7	0.21
Selenium	10.7	16.5	16.9	14.2	7*	0.01
Zinc	54.1	54.2	48.7	54.1	46.5*	0.03

ANOVA; Tukey test; adjusted for age and energy; *: Significantly higher proportion of low intake

Based on the presence of fractures due to bone frailty, there was no significant association with the intake of any of antioxidants investigated (data not shown). The same was true regarding the intake of macronutrients and micronutrients related to bone health (calcium, phosphorus, magnesium, vitamins D, K and A) [22,25].

In general, a greater proportion of individuals with overweight or obesity had low vitamin A intake (Table 7). There were no significant differences regarding the intake of the other antioxidants.

**Table 7:** Proportion of Brazilian adults with low antioxidant intake according to nutritional state

	Normality (%)	Overweight (%)	Obesity (%)	P
Vitamin A	89.8	94.6*	91.4*	0.04
Vitamin C	82.8	86.4	80.5	0.11
Vitamin E	99.8	99.8	99.7	0.29
Selenium	11.9	11.6	10.9	0.15
Zinc	43.1	47.8	46.7	0.22

ANOVA; Tukey test, adjusted for age and energy; *: Significantly higher proportion of low intake

## Discussion

The results of the present study demonstrate that, while there was a difference in dietary antioxidant intake between Brazilian men and women, mean vitamin A and vitamin E intake was below the reference values, regardless of gender, age, skin color, economic status, social class, nutritional state and region of the country.

Brazil has been following the worldwide tendency of an extended life expectancy and ageing of the population [27,28]. The low intake of antioxidants, especially among individuals over 60 years of age, can have serious public health consequences. In elderly individuals, besides the reduction in the capacity to prevent and remove oxidation products, there is also a greater possibility of generating reactive oxygen species and a lesser dietary antioxidant intake related to a series of factors inherent to ageing, such as a reduction in ingestion and the absorption capacity of the digestive tract [27,29]. Thus, the low intake identified in the BRAZOS study may be an indicator that antioxidant intake should be improved.

In the present study, Brazilian people had significantly lower intakes of antioxidants. This may be consequence of consumption underreporting. However, other factors can particularly affect the intake. The simple fact of living longer is associated with less functional abilities, and other conditions, i.e. being widowed and with children living geographically apart can reduce motivation for cooking. Financial issues, such as living on a fixed/low income, increased dependence on social security or pension may also contribute to a greater nutrient inadequacy [30].

Currently, obesity is considered the second greatest cause of avoidable death in modern society. According to data from the 2002-2003 Brazilian Family Budget Survey [2], 40.6% of the population is overweight, with greater prevalence among individuals between 50 and 70 years of age. The BRAZOS study reveals a similar frequency of overweight and obesity (57.7%) among men and women over 40 years of age [22,23]. Obesity and the consequent non-communicable chronic diseases stemming from it do not only affect the mortality rate, but are also associated to concomitant diseases that represent a huge economic burden on the healthcare system and society alike. Costs with care and medication are significantly higher for patients with excess weight, increasing with each unit of body mass index in the shape of a "J" curve [31].

In western societies, the increase in obesity and associated chronic disease has been related to an increase in calorie intake stemming from the choice of unbalanced diets, the intake of foods with a high energy density rich in fats and carbohydrates [32] as well as a progressive increase in the portions of food [6,32-34]. It should be stressed that the greater total food and calorie intake by obese individuals is not necessarily associated to the consumption of foods that are a source of micronutrients, as evidenced in the present study. In general, there is an insufficient intake of sources of vitamins and minerals, which, in the long run, could increase the risk of the development or aggravation of metabolic disorders.

Conversely, the relation between anthropometric nutritional state and food intake data or the concentrations of vitamins and minerals is controversial. A number of studies have found lower serum levels of carotenoids in obese individuals in comparison to individuals within the ideal weight range, with no significant difference regarding food intake [31,35-38]. Thus, greater serum inadequacy of these antioxidants is believed to be associated with a greater metabolic demand rather than oxidative stress in obese individuals [39].

Traditionally, vitamin A deficiency is related to alterations in visual acuity and the immune system. Its role as a modulator of fat tissue, causing greater adipogenesis, has recently been demonstrated [40]. Moreover, individuals with a lower plasma concentration of this vitamin may be at greater risk for cardiovascular mortality [41], as vitamin A participates in the prevention and slowing of the atherogenesis process through the inhibition of the oxidation of LDL-cholesterol and a reduction in the formation of foam cells [20,42]. The data from the present study corroborate previous studies carried out in Bambuí (Minas Gerais, Brazil) [43] and Ponta Porã (Mato Grosso do Sul, Brazil) [44] regarding the high proportion of individuals with low vitamin A intake (92.4%, 99.8% and 87.5%, respectively). The most likely explanation for this finding, even in regions with a large amount of food sources of this vitamin, such as the northern and northeastern regions, is more related to cultural and eating habits than economic factors [45,46]. According to the National Demographic Children's and Women's Health Survey [47], The greatest prevalence of vitamin A deficiency was found in the southeastern region and the lowest was found in the northern region.

Vitamin E is a lipophilic antioxidant in cell membranes and has considerable importance to the neutralization of peroxynitrites and protection from oxidative stress, particularly the sweeping away of free radicals [10]. This is the first study to evaluate the dietary intake of vitamin E among the Brazilian population. Two surveys in the United States found intakes of vitamin E superior to the values found in our study. The Third National Health and Nutrition Examination Survey (NHANES III 1988-94) reported a median intake for men and women (31 to 50 years old) of 11.7 mg and 9.1 mg, respectively, while the Continuing Survey of Food Intakes by Individuals (CSFII 1994-96) described lower median intake in this same age (9.3 mg for men and 6.8 mg for women) [9,48]. A British cohort study that evaluated vitamin intake at 4 and 43 years of age identified that those with the lower intake had higher risk for hypertension and higher waist circumference. In this study, the highest intake of vitamin E, both in childhood and adulthood, was found in women (57%) [49]. These findings are different from our results, which indicate similar intake in both genders. In another study, Morris et al found that higher intake of vitamin E, from both food and supplements, was associated with lesser change in cognitive score/year in elderly [50]. In our study, the mean intake was similar to the values reported for median intakes in the lower quintile.

In order to achieve the current recommendation for vitamin E intake proposed by the DRIs, it is necessary to consume large quantities of food sources of this vitamin, such as oils, oil-rich foods and whole-grain cereals [51,52], which are also a source of unsaturated fatty acids. Thus, a diet rich in fruit and vegetables, but with a low intake of these food sources, likely contains less than 15 mg of α-tocopherol. Some of these foods, such as nuts, are generally not part of Brazilian cultural habits. Soybean oil and margarine are the main sources of fats in Brazil [2], and vitamin E requirements increase as polyunsaturated fats intake increases. Furthermore, a diet rich in fats/ oils may not be of advantage due to their high-energy or caloric value [9]. The supplementation or fortification of foods with vitamin E may be an interesting strategy for improving dietary adequacy [53,54]. However, it also should be taken into consideration that vitamin E is a difficult element for assessing. Low intakes may be due to underreporting of energy and consequently of fat intake, its primary source, or difficulty in estimating amounts of oils and fats added to food preparation and actual amount absorbed into the cooked product [9].

Selenium is an essential component for the formation of glutathione peroxydase, which is an enzyme involved in the detoxification of hydrogen peroxide and lipid hydroperoxidation as well as an important co-enzyme for the synthesis of proteins related to the immune and neurophysiological systems [55]. In the BRAZOS study, we found a small proportion of individuals (13.4%) with a low intake of this micronutrient, even lower than that reported by Fernandes et al. (21.1%) in adults with metabolic syndrome [33]. However, caution should be exercised when analyzing this finding, as the amount of selenium in foods is related to its presence in the soil and the amount found in a given food source differs depending on its region of origin [56]. For instance, in the case of the Brazil nut, which is an excellent source of this mineral, Freitas et al. [57] identified contents ranging from 0.003 mg to 5.12 mg in 100 g (range of 1:17000). Moreover, studies have found that the states of São Paulo and Mato Grosso have lower concentrations of this nutrient in the soil [58]. In the present study, selenium intake was highest in the northern region, probably due to the greater consumption of fish and cashews.

More than half of the sample, especially those over 60 years of age (data not shown) had an insufficient zinc intake, which corroborates the findings described by Cozzolino [58]. Rats with zinc deficiency have a reduced tolerance to glucose with no alteration in insulin production in response to glucose injection [59]. Likewise, individuals with type II diabetes may have a lower serum level of zinc [60]. More recently, Marreiro et al. [61] found low plasma concentrations of zinc in obese individuals and demonstrated that the supplementation of this mineral reduces insulin resistance.

Vitamin C (ascorbic acid) is one of the most important hydrosoluble antioxidants. It is capable of inhibiting the formation of hydroxyl free radicals and protecting from the peroxidation of lipids and LDL by sequestering peroxyl radicals [62] in joint action with vitamin E [15]. It should be stressed that the proportion of individuals with low vitamin C and E intake may be underestimated, as the percentage of regular smokers was 25% [25], which is a condition that increases the need for these vitamins [63].

The present study has limitations that should be considered. Incomplete information about micronutrients in software's database should always be accounted for since the American nutritional reference database used does not include the regional peculiarities of some typical Brazilian foods. However, the researchers took care to adjust the nutritional aspects by means of a laboratory analysis of foods reported by the population, such as regional fruit, dishes, spices, delicacies, etc. Another important aspect is there was no extrapolation to other age groups and/or stages of life, as only individuals over 40 years of age were included. Although an adequate data collection method for the estimation of mean dietary intake [64], the evaluation of intake based on a single 24-hour recall does not reflect habitual or long-term intake and does not allow an analysis of intake intra-variability. Moreover, although mean nutrient intake is adequate for the description of intake among groups, it does not allow comparisons with reference values (EAR) for the identification of the prevalence of deficiencies. Other substances not evaluated in this study should be considered in further analyzes due to their role in modulating oxidative stress, such as copper, manganese, as well as that bioactive. Finally, plasma concentrations of the antioxidants were not determined, which does not allow ensuring the clinical outcome.

## Conclusion

The BRAZOS study revealed low dietary antioxidant intake in the adult Brazilian population, regardless of social class, economic status, region of the country or skin color. These data suggest that cultural habits and food choices seem to be the most determinant factors of the inadequate intake of micronutrients that are essential to overall health. The best medium and long-term public health strategy for improving the low dietary intake of nutrients is an adequate nutrition education policy in Brazil. Measures for fortifying foods and supplementation, in special cases, as well as the stimulus to guarantee on the availability of antioxidant-rich food, such as fruits and vegetables, could also have a substantial impact on minimizing inadequate nutrient intake. However, prospective studies are needed to demonstrate the association of these findings with a greater risk of non-communicable chronic diseases, especially obesity and osteoporosis.

## Competing interests

This study was supported by a grant from Wyeth Consumer Healthcare.

## Authors' contributions

MMP - has made substantial contributions to acquisition of data, analysis and interpretation of data also have been involved in drafting the manuscript. RMC - has made substantial contributions to acquisition of data, analysis and interpretation of data also have been involved in drafting the manuscript. GVC - has made substantial contributions to conception and design of data and also analysis and interpretation of data. LA - has been involved in drafting the manuscript or revising it critically for important intellectual content. CRJ - has been involved in drafting the manuscript, made substantial contributions to conception and design and revising it critically for important intellectual content. PSG - has been involved in training of interviewers on nutritional issues and revising final version of the manuscript. MBF - has made substantial contributions to conception and design, have been involved in drafting the manuscript and also revising it critically for important intellectual content and have given final approval of the version to be published. All authors read and approved the final manuscript.

## Authors' information

**Marcelo M Pinheiro: **Assistant-Doctor, Rheumatology Sector, Federal University of Sao Paulo/ Paulista School of Medicine (Unifesp/ EPM), Brazil **Rozana M Ciconelli: **Assistant-Doctor, Paulista Center for Health Economics, Unifesp/ EPM, Brazil

**Gabriela Villaça Chaves: **Master in Internal Medicine, Doctoral student in Internal Medicine, Researcher at the Micronutrient Research Center, Josué de Castro Nutrition Institute, Federal University of Rio de Janeiro, Brazil

**Luana Aquino: **Master in Human Nutrition, Researcher at the Micronutrient Research Center, Josué de Castro Nutrition Institute, Federal University of Rio de Janeiro, Brazil

**Claudia R Juzwiak: **Doctor of Science, Adjunct Professor, Department of Health, Education and Society, Nutrition Course, Unifesp/Baixada Santista Campus, Brazil

**Patrícia Souza Genaro: **Doctor in Public Health, Post-Doc student, Rheumatology Division, Federal University of Sao Paulo, Unifesp/ EPM, Brazil

**Marcos Bosi Ferraz: **Adjunct Professor and Coordinator of the Paulista Center for Health Economics, Unifesp/ EPM, Brazil

## References

[B1] Instituto Brasileiro de Geografia e Estatística 2000 http://www.ibge.gov.br/home/estatistica/populacao/censo2000/default.shtm

[B2] Instituto Brasileiro de Geografia e Estatística Pesquisa de orçamentos familiares 2002-2003: Análise da disponibilidade domiciliar de alimentos e do estado nutricional no Brasil 2004 Rio de Janeiro: Ministério do Planejamento e Orçamento/IBGE

[B3] Shetty P Shimidhuber J Introductory lecture the epidemiology and determinants of obesity in developed and developing countries Int J Vitam Nutr Res 2006 4 157 62 10.1024/0300-9831.76.4.157 17243077

[B4] [WHO]. World Health Organization The world report 2002: reducing risks, promoting healthy life 2002 Geneve: World Health Organization

[B5] [WHO] World Health Organization Prevention of cardiovascular disease: pocket guidelines for assessment and management of cardiovascular risk 2007 Geneve

[B6] Riccioni G Bucciarelli T Mancini B Corradi F Di Ilio C Mattei PA D'Orazio N Antioxidant vitamin supplementation in cardiovascular diseases Ann Clin Lab Sci 2007 37 89 95 17311876

[B7] Department of Health and Human Services National Center for Health Statistics. Healthy people 2000: national health promotion and disease prevention objectives 1991 Washington

[B8] [IOM] Institute of Medicine Dietary Reference Intakes for thiamin, riboflavin, niacin, vitamin B6, folate, vitamin B12, panthotenic acid 1998 Washington, D.C.: National Academy Press 23193625

[B9] [IOM] Institute of Medicine Dietary reference intakes. Vitamin C, Vitamin E, Selenium, and Carotenoids 2000 Washington, D.C.: National Academy Press 25077263

[B10] [IOM] Institute of Medicine Dietary reference intakes. Vitamin A, Vitamin K, Arsenic, Boron, Chromium, Copper, Iodine, Iron, Manganese, Molybdenum, Nickel, Silicon, Vanadium, and Zinc 2001 Washington, D.C.: National Academy Press 25057538

[B11] Després JP Pascot A Lemieux I Risk factors associated with obesity: a metabolic perspective Ann Endocrinol 2000 61 Suppl 6 31 38 11148334

[B12] Schaefer EJ Lipoproteins, nutrition, and heart disease Am J Clin Nutr 2002 75 191 212 11815309 10.1093/ajcn/75.2.191

[B13] Halliwell B Aeschbach R Löliger J Aruoma OI The characterization of antioxidants Food Chem Toxicol 1995 33 601 17 10.1016/0278-6915(95)00024-V 7628797

[B14] Berger MM Can oxidative damage be treated nutritionally? Clin Nutr 2005 24 172 83 10.1016/j.clnu.2004.10.003 15784476

[B15] Clark SF The biochemistry of antioxidants revisited Nutr Clin Pract 2002 17 5 17 10.1177/011542650201700105 16214960

[B16] Jones DP Redefining oxidative stress Antioxid Redox Signal 2006 8 9-10 1865 79 10.1089/ars.2006.8.1865 16987039

[B17] Sies H Biological redox systems and oxidative stress Cell Mol Life Sci 2007 64 17 2181 8 10.1007/s00018-007-7230-8 17565441 PMC11136165

[B18] Czernichow S Hercberg S Interventional studies concerning the role of antioxidants vitamins in cardiovascular diseases; a review J Nutrition Health & Aging 2001 5 188 195 11458291

[B19] Rissamen TH Voutilainem S Nyyssonen K Salonen R Kaplan GA Salonen JT Serun lycone concentrations and carotid atherosclerosis: the Kuopio ischaemic heart disease risk factor study Am J Clin Nutr 2003 77 133 38 12499332 10.1093/ajcn/77.1.133

[B20] Sesso HD Buring JE Norkus EP Gaziano JM Plasma lycopene, other carotenoids, and retinol and the risk of cardiovascular disease in women Am J Clin Nutr 2004 79 47 53 14684396 10.1093/ajcn/79.1.47

[B21] National Home Sampling Survey (PNAD) 2003 http://www.ibge.gov.br/home/estatistica/populacao/trabalhoererendimento/pnad2003/coeficiente_brasil.shtm

[B22] Pinheiro MM Scuch NJ Genaro PS Ciconelli RM Ferraz MB Martini LA Nutrient intakes related to osteoporotic fractures in men and women - The Brazilian Osteoporosis Study (BRAZOS) Nutr J 2009 8 6 10.1186/1475-2891-8-6 19178745 PMC2646749

[B23] [WHO] World Health Organization Obesity: preventing and managing the global epidemic. Report of WHO consultation on obesity 1998 World health organization, Geneva 11234459

[B24] Nutrition Coordinating Center (NCC), University of Minnesota Nutrition Data System for Research-NDS-R [programa de computador] 2005 University of Minnesota: Minneapolis

[B25] Pinheiro MM Ciconelli RM Martini LA Ferraz MB Clinical risk factors for osteoporotic fractures in Brazilian women and men: The Brazilian Osteoporosis Study (BRAZOS) Osteoporos Int 2009 20 399 408 10.1007/s00198-008-0680-5 18597037

[B26] Willett W Stampfer M Willett W Implications of total energy intake for epidemiological analyses Nutritional epidemiology 1998 2 New York: Oxford University Press 273 301

[B27] Campos MTFS Monteiro JBR Ornelas APRC Fatores que afetam o consumo alimentar e a Nutrição do idoso Rev Nutr Campinas 2000 13 157 165

[B28] Lebrão ML Laurenti R Saúde, bem-estar e envelhecimento: o estudo SABE no municipio de São Paulo Rev Bras Epidemiol 2005 8 127 41

[B29] Beckman KB Ames BN The free radical theory of aging matures Physiol Rev 1998 78 547 81 9562038 10.1152/physrev.1998.78.2.547

[B30] Chernoff R Micronutrients requirements in older women Am J Clin Nutr 2005 81 suppl 1240S 5S 15883458 10.1093/ajcn/81.5.1240

[B31] Ancona-Lopez F Juzwiak CR Guiné RP Social impact of modern diet and tendencies Food, diet, health: past, present and future tendencies 2010 Hanppange, New York: Nova Science Publishers

[B32] Young LR Nestle M The contribution of expanding portion sizes to the US obesity epidemic Am J Public Health 2002 92 246 9 10.2105/AJPH.92.2.246 11818300 PMC1447051

[B33] Fernandes M Paes C Nogueira C Souza G Aquino L Borges F Ramalho A Perfil de consumo de nutrientes antioxidantes em pacientes com síndrome metabólica Rev Ciênc Méd 2007 16 209 1921426420

[B34] Neumann AI Martins IS Marcopito LF Araujo EA Dietary patterns associated with risk factors for cardiovascular disease in a Brazilian city Rev Panam Salud Publica 2007 22 329 39 10.1590/S1020-49892007001000006 18198042

[B35] Neuhouser ML Rock CL Eldridge AL Serum concentrations of retinol, alfa-tocoferol and carotenoids are influenced by diet, race and obesity in a sample of health adolescents J Nutr 2001 131 2184 219 11481415 10.1093/jn/131.8.2184

[B36] Sarni RS Kochi C Vitamin A: blood level and dietetics intake in stunted children and adolescents without hormonal disease Rev Assoc Med Bras 2002 48 48 53 10.1590/S0104-42302002000100032 12185636

[B37] Sarni RS Kochi C Impact of vitamin A megadose supplementation on the anthropometry of children and adolescents with non-hormonal statural deficit: a double-blind and randomized clinical study Int J Vitam Nutr Res 2003 73 303 11 10.1024/0300-9831.73.4.303 12951904

[B38] Silva LSVL Veiga GVV Ramalho RA Association of serum concentrations of retinol and carotenoids with overweight in children and adolescents Nutrition 2007 23 392 97 10.1016/j.nut.2007.02.009 17433621

[B39] Matsuoka H Endothelial dysfunction associated with oxidative stress in human Diab Res Clin Pract 2001 54 65 72 10.1016/s0168-8227(01)00337-0 11733111

[B40] Jeyakumar SM Vajerswaria A Giridharan NV Chronic dietary vitamin A supplementation regulates obesity: in obese mutant rat model of WNIN/Ob strain. Obesity research J Mol Endocrinol 2006 35 391 98 10.1038/oby.2006.7 16493122

[B41] Ochoa JJ Vilchez MJ Mataix J Ibáñez-Quiles S Palacios MA Muñoz-Hoyos A Oxidative stress in patients undergoing cardiac surgery: comparative study of revascularization and valve replacement procedures J Surg Res 2003 111 248 54 10.1016/S0022-4804(03)00106-9 12850470

[B42] Delport R Antioxidants and coronary artery disease risk in South African males Clinical Chimical Acta 1998 278 55 60 10.1016/S0009-8981(98)00131-4 9877124

[B43] Lopes ACS Caiaffa WT Sichieri R Mingoti SA Lima-Costa MF Consumo de nutrientes em adultos e idosos em estudo de base populacional: Projeto Bambuí Cad Saúde Pública 2005 21 1201 09 16021257 10.1590/s0102-311x2005000400022

[B44] Fietz VR Estado nutricional, consumo de alimentos e condições socioeconômicas das famílias de assentamento rural em Mato Grosso do Sul PhD thesis 2007 Faculdade de Engenharia de Alimentos. Universidade de Campinas

[B45] Ramalho RA Flores H Accioly E Saunders C Vitamin A Deficiency in Brasil Revista da Sociedad Iberoamericana de Información Científica 2005 1 1 9

[B46] Ramalho RA Diversificação Alimentar como parte de um Programa de Combate à Deficiência de Vitamina A Nutrição em Pauta 2008 88 5 921480979

[B47] Ministério da Saúde Pesquisa Nacional de Demografia da Saúde da Crianças e da Mulher http://bvsms.saude.gov.br/bvs/pnds/index.php

[B48] Dixon LB Winkleby MA Radimer KL Dietary intakes and serum nutrients differ between adults from food-insufficient and food-sufficient families: Third National Health and Nutrition Examination Survey, 1988-1994 J Nutr 2001 131 4 1232 46 11285332 10.1093/jn/131.4.1232

[B49] Mishra GD Malik NS Paul AA Wadsworth MEJ Bolton-Smith C Childhood and adult dietary vitamin E intake and cardiovascular risk factor in mid life in the 1946 British Birth Cohort Eur J Clin Nutr 2003 57 1418 25 10.1038/sj.ejcn.1601706 14576755

[B50] Morris MC Evans DA Bienias JL Tangney CC Wilson RS Vitamin E and cognitive decline in older persons Arch Neurol 2002 59 1125 32 10.1001/archneur.59.7.1125 12117360

[B51] Horwitt MK Critique of the requirement for vitamin E Am J Clin Nutr 2001 73 1003 5 11382651 10.1093/ajcn/73.6.1003

[B52] Traber MG Vitamin E: too much or not enough? Am J Clin Nutr 2001 73 987 98 10.1093/ajcn/73.6.997 11382648

[B53] Beitz R Mensink GBM Fischer B Thamm M Vitamins dietary intake and intake from dietary supplements in Germany Eur J Clin Nutr 2002 56 539 45 10.1038/sj.ejcn.1601346 12032654

[B54] Sichert-Hellert W Kersting M Manz F Changes in time-trends of nutrient intake from fortified and non-fortified food in German children and adolescents: 15 year results of the DONALD Study Eur J Nutr 2001 40 49 55 10.1007/PL00007385 11518199

[B55] Navarro-Alarcon M Cabrera-Vique C Selenium in food and the human body: a review Sci Total Environ 2008 400 115 41 10.1016/j.scitotenv.2008.06.024 18657851

[B56] Gonzaga IB Martens A Cozzolino MF Biodisponibilidade de nutrientes 2005 São Paulo: Manole

[B57] Freitas SC Gonçalves EB Meta-análise do teor de selênio em castanha-do-brasil Braz J Food Technol 2008 11 54 62

[B58] Cozzolino SMF Deficiências de minerais Estudos Avançados 2007 21 119 2614666246

[B59] Boquist L Lernmark A Effects on the endocrine pancreas in Chinese Hamsters fed zinc deficient diets Acta Pathol Microbiol Scand 1969 6 215 10.1111/j.1699-0463.1969.tb03252.x 4906988

[B60] Soinio M Marniemi J Laakso M Pyörälä K Lehto S Rönnemaa T Serum zinc level and coronary heart disease events in patients with type 2 diabetes Diabetes Care 2007 30 523 8 10.2337/dc06-1682 17327315

[B61] Marreiro DN Fisberg M Cozzolino SM Zinc nutritional status and its relationships with hyperinsulinemia in obese children and adolescents Biol Trace Elem Res 2004 100 137 49 10.1385/BTER:100:2:137 15326363

[B62] Shills ME Olson JA Shike M Ross AC Modern Nutrition in Health and Disease 1999 Dona Balado

[B63] Madruga de Oliveira A Rondó PH Barros SP Concentrations of ascorbic acid in the plasma of pregnant smokers and non-smokers and their newborns Int J Vitam Nutr Res 2004 74 193 8 10.1024/0300-9831.74.3.193 15296078

[B64] Hoffmann K Boeing H Dufour A Volatier JL Telman J Virtanen M Estimating the distribution of usual dietary intake by short-term measurements Eur J Clin Nutr 2002 56 53 62 10.1038/sj.ejcn.1601429 12082518

